# Cystatin C as a Candidate Biomarker of Cardiovascular Outcomes: Too Near, but too Far from Reality

**DOI:** 10.5935/abc.20180226

**Published:** 2018-12

**Authors:** Luiz Sérgio F. de Carvalho, Thiago Quinaglia AC Silva, Otávio Rizzi Coelho-Filho

**Affiliations:** 1 Disciplina de Cardiologia - Departamento de Medicina Interna - Universidade Estadual de Campinas (UNICAMP), Campinas, SP - Brazil; 2 Escola Superior de Ciências da Saúde, Brasília, DF - Brazil

**Keywords:** Cardiovascular Diseases, Cystatin C, Biomarkers, Atherosclerosis, Glomerular Filtration Rate

While the development of novel risk factors for cardiovascular risk assessment is necessary to improve risk stratification, proving its clinical value on top of traditional risk factors is routinely challenging.^1-3^ Besides all the innovative and straightforward biomarker research published in the last decades, only very few markers of cardiovascular risk have shown clinical significance.^4,5^ Among many of them, cystatin C has emerged some years ago as a candidate for improving cardiovascular risk stratification.

In the *Cardiovascular Health Study* (CHS),^6^ a community-based and longitudinal study with over 4,600 elderly individuals, cystatin C has shown to predict cardiovascular outcomes. As compared with the lowest quintile, the highest quintile of cystatin C was associated with a significantly increased risk of death from cardiovascular causes (hazard ratio [HR] 2.27 [1.73 to 2.97]), myocardial infarction (HR 1.48 [1.08 to 2.02]), and stroke (HR 1.47 [1.09 to 1.96]) after multivariate adjustment. However, cystatin C is typically known as a marker of renal function, being roughly correlated with glomerular filtration rate in early stages of kidney diseases.^7,8^ Reasonably, since glomerular function is a strong surrogate marker of cardiovascular disease, it suggests an obvious association between cystatin C and cardiovascular outcomes. A mechanism to avoid the impact of this inexorable bias was to study only individuals with normal kidney function. Yet, additional studies have shown inconsistent magnitudes of effect between cystatin C and cardiovascular outcomes.

In that context, Einwoegerer and Domingueti^9^ in this issue of the *Brazilian Archives of Cardiology* investigated the role of plasma cystatin C levels on the risk of all-cause mortality and other softer endpoints by pooling studies of individuals with normal renal function. Unfortunately, only two studies compared quartiles of cystatin C with multivariate regression analysis, hence providing a sample size that is not too far from the original *Ludwigshafen Risk and Cardiovascular Health* (LURIC) study.^10^ The meta-analysis suggested a robust association between high levels of cystatin C and the risk of all-cause mortality in individuals with normal renal function (HR 2.28 [1.70 - 3.05], p < 0.001). Heterogeneity among studies was substantial (I^2^ > 50%) and no sensitivity analysis was provided. Besides the critical limitations in meta-analysis data, authors also provided substantial elements in a systematic review of studies on the same topic.

Although a first step for a candidate biomarker is to show strong association with a clinical outcome, this is not sufficient to prove its complementary clinically usefulness beyond traditional cardiovascular risk factors, such as age, gender, smoking, hypertension, diabetes, hyperlipidemia, obesity and aortic stenosis. A next fundamental step is to show whether cystatin C could improve risk prediction of cardiovascular outcomes in Receiver operating characteristic (ROC) curves models, net reclassification index (NRI) and integrated discrimination index (IDI) compared-to or added-to the Framinghan Heart Risk, ASCVD risk score, or any validated cardiovascular risk scores/engines.^11,12^

Besides the potential mechanistic link between cystatin C and atherosclerotic disease, this association is unlikely to be causal. By using a Mendelian randomization approach, which takes into account both the genetic association with cystatin C and CVD to triangulate the causal effect, and combining a set of cohorts of over 250,000 individuals with 63,000 cases of cardiovascular events from the *Cystatin C Mendelian Randomization Consortium* no association could be found.^13^ This finding in no way suggests that we should abandon the use of cystatin C for risk stratification purposes in kidney diseases, but there are two key messages in it: (i) it alerts against the chase of therapeutic strategies that target at lowering plasma cystatin C levels; (ii) it also indicates a low likelihood of association between cystatin C as a surrogate cardiovascular marker on top of classical risk factors. However, the last word in favor or against the use of cystatin C in clinical practice for cardiovascular risk stratification of individuals with normal renal function should be based on studies evaluating detrimental effects of this marker on established risk scores/engines.
